# The Neuroprotective Efficacy of Postnatal Magnesium Sulfate in Term or Near-Term Infants With Moderate-to-Severe Birth Asphyxia

**DOI:** 10.7759/cureus.16826

**Published:** 2021-08-02

**Authors:** Nadeem Iqbal, Javaria Younus, Muneeba Malik, Bushra Fatima, Ahmed Imran, Shazia Maqbool, Khawaja Ahmad Irfan Waheed, Khalid Haque

**Affiliations:** 1 Neonatology, The Children’s Hospital & The Institute of Child Health, Lahore, PAK; 2 Developmental and Behavioural Pediatrics, The Children’s Hospital & The Institute of Child Health, Lahore, PAK; 3 Pediatric Radiology, The Children’s Hospital & The Institute of Child Health, Lahore, PAK

**Keywords:** neuroprotection, neurodevelopment, hie, magnesium sulfate, neonates

## Abstract

Background

In Pakistan, the neonatal mortality rate is 41 per 1,000 live births and birth asphyxia is one of the leading causes of neonatal mortality and morbidity. The goal of this study was to determine whether postnatal magnesium sulfate therapy can improve short- and long-term neurological outcomes in term or near-term neonates with moderate-to-severe birth asphyxia.

Methodology

This prospective double-blind randomized controlled trial was conducted in the Neonatology Department of the Children’s Hospital & The Institute of Child Health, Lahore. A total of 62 neonates (31 in each group) were randomized to receive either three doses of magnesium sulfate infusion at 250 mg/kg per dose, 24 hours apart (treatment group), or three doses of injection 10% distilled water infusion at 3 mL/kg, 24 hours apart (placebo group). Both groups received similar supportive care. The neurodevelopmental assessment was done at six months of age using the ShaMaq Developmental Inventory.

Results

Demographic data such as gestational age, mean weight, age at presentation, gender, hypoxic-ischemic encephalopathy grade, mode of delivery, and the presence of seizures at presentation were comparable between both groups. In the magnesium sulfate group, statistically significant results were seen in terms of early seizure control (p = 0.001), early initiation of feed (p = 0.002), and shorter duration of hospital stay (p = 0.003). Moreover, the magnesium sulfate group had lower mortality compared to the control group, though it was not statistically significant (p = 0.390). There was no significant difference in terms of cranial ultrasound findings between the two groups (p = 0.783) at the time of discharge. Regarding the neurodevelopmental delay, there was no significant difference between the magnesium sulfate and control groups (p = 0.535).

Conclusions

Postnatal magnesium sulfate treatment improves short-term neurologic outcomes at discharge in term or near-term neonates with moderate-to-severe perinatal asphyxia. However, no difference was noted in the neurodevelopmental outcome at six months.

## Introduction

Birth asphyxia is one of the leading causes of neonatal morbidity and mortality globally [1,2]. In Pakistan, the neonatal mortality rate is 41.22 per 1,000 live births, with birth asphyxia contributing to 20.9% of neonatal deaths [3]. Among neonates with moderate hypoxic-ischemic encephalopathy (HIE), 10-20% die and 30-40% develop neurological complications, whereas 50-90% of neonates with severe HIE die, with survivors developing neurological deficits [4,5].

In HIE, brain injury develops in two phases [6]. In the early phase, there is increased release along with decreased uptake of glutamate, an excitatory neurotransmitter that acts on N-methyl-D-aspartate (NMDA) receptors, which are postsynaptic ion channels in the brain. They open NMDA channels leading to the increased entry of calcium into the neuronal cell causing irreversible damage to the neurons. NMDA receptor antagonists such as magnesium block the entry of calcium ions into the cell and preserve neuronal structure and function [7]. Magnesium sulfate gates the NMDA channels in a voltage-dependent manner and prevent brain injury [8]. Thus, magnesium has been proposed to prevent brain injury [9]. In a systematic review and meta-analysis, Tagin et al. [10] reported an improvement in the short-term outcome with the use of magnesium in HIE. Additionally, they reported the absence of significant side effects and encouraged the need for further studies to determine the long-term benefits of magnesium.

In moderate birth asphyxia, therapeutic hypothermia reduces morbidity and mortality; however, it has limited efficacy in severe birth asphyxia [11]. Therefore, further research is required to find an adjuvant therapy [12,13]. This study was conducted to determine whether postnatal magnesium sulfate therapy could improve short- and long-term neurological outcomes in term or near-term neonates with moderate-to-severe birth asphyxia.

## Materials and methods

This prospective, double-blind, randomized controlled study (RCT) was conducted in the Neonatology Department of The Children’s Hospital & The Institute of Child Health, Lahore from January to June 2020. The study was initiated after obtaining permission from the Institutional Review Board of The Children’s Hospital & The Institute of Child Health, Lahore. Data were collected on a predesigned proforma after obtaining informed consent from the parents or guardians of the patient. The sample size was calculated to be 62 using the World Health Organization’s software, namely, “Sample size determination for health studies,” using 90% power, 5% significance level, and by considering efficacy as outcomes of 77% and 37% in the magnesium sulfate and placebo groups, respectively, based on a previous study [14]. Additionally, a 20% loss to follow-up was also considered in determining the sample size. A consecutive probability sampling technique was used. Neonates with a gestational age of >35 weeks, <24 hours of age at presentation, and an inability to initiate or sustain breathing at birth along with clinical features suggestive of encephalopathy manifesting as neurological depression, depressed respiratory drive, and seizures were included in the study. Neonates were classified into moderate or severe HIE according to the modified Sarnat and Sarnat staging. Neonates with sepsis, pneumonia, congenital heart disease, an inborn error of metabolism, or congenital malformations were excluded from the study. Qualitative variables included age at presentation, weight, gestational age, initiation of feed, seizure control, and duration of stay, whereas gender, HIE staging, seizures at presentation, cranial ultrasound findings, mortality, and neurodevelopmental status were quantitative variables.

The short-term outcome was measured in terms of early initiation of feed, early seizure control, duration of stay, outcome (discharge/death), and normal/abnormal cranial ultrasound findings at discharge. Cranial ultrasound was performed by the same sonologist. The six-month outcome was measured in terms of neurodevelopmental assessment (normal/delayed) using the ShaMaq Developmental Inventory.

Neonates were randomized equally to both groups (magnesium sulfate and placebo) through the lottery method. A randomization slip was picked and attached to the neonate’s file by the pharmacist for every neonate included in the study. Similar supportive treatment was provided to all neonates. The treatment group received magnesium sulfate infusion at 250 mg/kg/dose in 10% dextrose to make volume equal to 3.0 mL/kg/dose given over one hour, with two additional doses repeated at 24 and 48 hours. The placebo group received 10% dextrose 3.0 mL/kg/dose over one hour, with two additional doses repeated at similar intervals. The doctor and the nurse were blinded while the pharmacist was responsible to break the code in case of an adverse event.

Results were analyzed using SPSS version 20 (IBM Corp., Armonk, NY). Qualitative variables were analyzed using frequency and percentages. The chi-square test was used for statistical analysis. Quantitative variables were assessed using mean and standard deviation, and a t-test was used for statistical analysis. A p-value of ≤0.05 was considered statistically significant.

## Results

**Figure 1:**
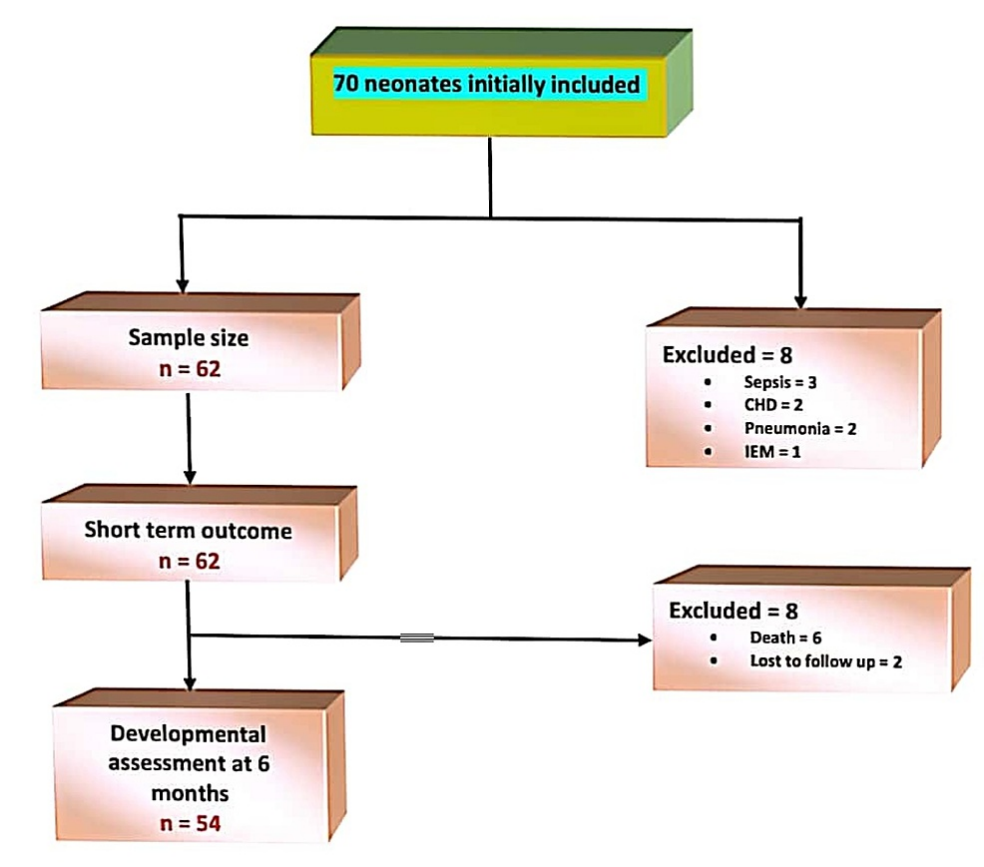
Flowchart showing the number of study participants included and excluded from the analysis. CHD: congestive heart disease; IEM: inborn error of metabolism

A total of 70 neonates were initially included in the study; however, eight neonates were excluded after being diagnosed with sepsis, pneumonia, congenital heart disease, and an inborn error of metabolism. Finally, data were collected for 62 neonates who were included in the study (Figure 1).

Demographic data including mean birth weight, gestational age, mode of delivery, age at presentation, HIE stage, and the number of patients having seizures at presentation were comparable between the two groups (Table 1).

**Table 1:** Demographic data of the study participants. HIE: hypoxic-ischemic encephalopathy

	Magnesium sulfate group	Placebo group	P-value
Gender			0.576
Male	23	21	
Female	8	10	
Gestational age (weeks)	37.774 ± 0.762	37.548 ± 0.767	0.806
Mean weight (kg)	3.032 ± 0.253	3.125 ± 0.280	0.489
Age at presentation (hours)	8.612 ± 2.962	8.451 ± 3.096	0.774
HIE			0.490
Moderate	27	25	
Severe	4	6	
Mode of delivery			1.00
Spontaneous vaginal delivery	17	17	
Cesarean delivery	14	14	
Seizures at presentation			0.783
Yes	22	21	
No	9	10	

In the magnesium and placebo groups, seizures were controlled in 1.708 ± 0.464 days and 2.652 ± 1.112 days, respectively (p = 0.001). Feeding was commenced in 1.580 ± 0.564 days and 2.516 ± 0.961 days in the magnesium and placebo groups, respectively (p = 0.002). The mean duration of stay was 3.258 ± 1.063 days in the magnesium group compared to 4.387 ± 1.994 days in the placebo group (p = 0.003). The magnesium group had lower mortality compared to the placebo group (two deaths versus four deaths); however, it was not statistically significant (p = 0.390). In the magnesium group, 12 of 28 (42.85%) patients had an abnormal cranial ultrasound compared to 17 of 28 (60.71%) in the placebo group (p = 0.783). Abnormal cranial ultrasound findings included diffuse or focal echogenicity and intracranial hemorrhage (Table 2).

At the six-month follow-up, the long-term neurodevelopmental assessment revealed that six (22.2%) patients were developmentally delayed in the magnesium group compared to eight (29.6%) in the placebo group.

**Table 2:** Outcome variables of the study participants.

	Magnesium sulfate group	Placebo group	P-value
Seizure control (days)	1.708 ± 0.464	2.652 ± 1.112	0.001 (<0.05)
Initiation of feed (days)	1.580 ± 0.564	2.516 ± 0.961	0.002 (<0.05)
Duration of stay (days)	3.258 ± 1.063	4.387 ± 1.994	0.003 (<0.05)
Mortality	2	4	0.390
Abnormal cranial ultrasonography	12	17	0.783
Developmental status			0.535
Normal	21	19	
Delayed	6	8	

## Discussion

Pakistan is one of the countries with the highest neonatal mortality, with birth asphyxia being one of the major contributing factors [3]. Approximately 50-60% of neonates suffering from severe perinatal asphyxia develop encephalopathy [15]. In addition, perinatal asphyxia contributes to significant morbidity and neurodevelopmental delay among the survivors [1,2].

Although therapeutic hypothermia has shown improvement in neonates suffering from moderate birth asphyxia, its effectiveness is limited in severe birth asphyxia [11]. Therefore, further research is needed to identify adjuvant therapies for neuroprotection. Magnesium sulfate protects brain injury in neonates with birth asphyxia by gating the NMDA channels [8,9]. Many studies have been conducted to determine the role of magnesium in asphyxiated newborns.

Bhat et al. [14] conducted an RCT to evaluate the role of magnesium sulfate in neonates with perinatal asphyxia and demonstrated better oral feeding at discharge in the magnesium compared to the control group. This study is comparable to our study which showed statistically significant results with early initiation of feed in the magnesium group. In a study conducted on the role of magnesium sulfate in neonates with severe birth asphyxia by Ichiba et al. [16], there was no significant difference in early seizure control between the placebo and treatment groups; however, statistically significant results were observed in our study. In a systematic review and meta-analysis, Tagin et al. [10] reported a trend toward an increase in mortality in the magnesium group; however, our study showed that the magnesium group had lower mortality compared to the control group. This difference might be attributed to the variation in the number of doses of magnesium in some studies included in their meta-analysis.

In an RCT performed by Bhat et al., the radiological assessment demonstrated abnormal radiological findings in fewer neonates in the treatment group compared to the placebo group [14]. The results are comparable to the ultrasound findings of our study; however, the results of both studies are statistically insignificant. This can be attributed to the fact that MRI (diffusion-weighted images) is more sensitive and specific in detecting abnormal neuroimaging results in HIE, which was not used in either study. We could not perform MRIs for our patients because of cost limitations.

Gathwala et al. [17] evaluated the role of magnesium sulfate in severely asphyxiated neonates and concluded that fewer neonates were developmentally delayed in the treatment group compared to the placebo group. However, the difference was not significant. Similar results were obtained in our study, possibly because both studies were performed among the Asian population and developmental evaluation was performed at six months of age.

The strengths of this study include the evaluation of clinically important outcomes including neurodevelopmental outcome assessment at six months of age. Moreover, this study is a placebo-controlled, double-blinded RCT with sufficient power and larger sample size. The imitation of this study is that it does not separately delineate the effect of magnesium sulfate in moderate and severe HIE.

Further studies with long-term follow-up should be conducted to determine a stronger relationship of magnesium sulfate therapy with neurodevelopmental outcomes. The neuroprotective efficacy of magnesium sulfate therapy in neonates with moderate asphyxia needs to be ascertained and compared with magnesium sulfate therapy in neonates with severe asphyxia and placebo. Moreover, the appropriate timing of magnesium sulfate administration to obtain maximum efficacy needs to be determined.

## Conclusions

Postnatal magnesium sulfate therapy appears to be neuroprotective in term or near-term neonates with moderate-to-severe birth asphyxia as shown by early seizure control, shorter duration of hospital stay, and early initiation of feeding when magnesium sulfate was administered to the affected neonates within the first 24 hours of life. However, no improvement was seen in mortality, cranial ultrasonography changes at discharge, and neurodevelopmental outcome at six months of age.
